# Variant of Type 1B Duplicated Urethra in a Distal Hypospadiatic Boy: A Case Report

**DOI:** 10.7759/cureus.91120

**Published:** 2025-08-27

**Authors:** Turker Altuntas, Onur Can Ozkan, Selcuk Yucel, Kamil Cam, Tufan Tarcan, Cagri Akin Sekerci

**Affiliations:** 1 Department of Urology, Marmara University, School of Medicine, Istanbul, TUR; 2 Department of Urology, Pediatric Urology Division, Marmara University, School of Medicine, Istanbul, TUR; 3 Department of Urology, Koç University School of Medicine, Istanbul, TUR

**Keywords:** augmentation urethroplasty, distal hypospadias, duplicated urethra, pediatric urology, urethral duplication

## Abstract

We aimed to present a case of urethral duplication detected perioperatively and to discuss the management of this anomaly. Distal repair was planned for a one-year-old male patient presenting with hypospadias. Intraoperatively, a previously unreported variant of type 1B urethral duplication (Effmann classification) was identified for the first time. Augmentation urethroplasty using a preputial onlay flap was performed to anastomose the blind-ending ventral urethra with the functional urethra, followed by completion of hypospadias repair. Management of urethral duplication should be individualized based on the type of duplication and the patient’s symptoms. Diagnostic evaluation may include cystourethroscopy, voiding cystourethrography, retrograde urethrography, and ultrasonography to detect associated anomalies. Although dorsal accessory urethral excision is the most commonly performed procedure, there is no consensus on the optimal surgical approach. Asymptomatic urethral duplication may be detected incidentally during hypospadias surgery. In our case, a type 1B variant was managed with augmentation urethroplasty. Considering the planning and intraoperative findings of this case, even distal hypospadias repairs require comprehensive evaluation and should be performed in experienced centers capable of managing additional congenital anomalies.

## Introduction

Urethral duplication is a rare congenital anomaly that occurs more frequently in males [1]. Clinical findings vary depending on the existing anatomical variations. Duplication may present as a simple accessory urethral canal ending incompletely or as a completely separate second urethra originating independently from the bladder [2]. It most commonly occurs in the sagittal plane (dorsal and ventral), while horizontal duplications, where the urethras are positioned side-by-side (parallel), are much rarer [3]. Horizontal duplication may occasionally be associated with penile or bladder duplication [1]. Although the classification system proposed by Effmann et al. is widely accepted as the most anatomically and clinically comprehensive, it lacks the ability to clearly differentiate between sagittal and coronal collateral forms of duplication [3].

Although rarely reported in the literature, it should be kept in mind that patients presenting with hypospadias may have a duplicated urethra. Additionally, it should be noted that patients with a hypospadiac duplicated urethra may also have associated congenital anomalies, such as a posterior urethral valve, patent urachus, or renal ectopia [4]. Adults with a history of childhood surgery for hypospadias may present later in life with urinary tract infections or obstructive symptoms. In such cases, the possibility of a duplicated urethra should be considered [5].

The classification proposed by Effmann et al. is as follows: type I: blind-ending, incomplete urethral duplication or accessory urethra; type IA: typically distal in location; the accessory urethra opens on the dorsal or ventral surface of the penis but does not communicate with the primary urethra or the bladder; type IB: proximal type, which can be difficult to distinguish from a urethral diverticulum or Cowper’s ducts; the accessory urethra originates from the main urethral channel and ends blindly in the periurethral tissues; type II: complete, patent urethral duplication; type IIA: two separate external meatuses; type IIA1: two noncommunicating urethras, each arising independently from the bladder; type IIA2: the second urethral channel branches off from the first and courses independently to a second external meatus; type IIB: a single external meatus; type IIB1: a rare form in which two urethras arise either from the bladder or posterior urethra and join distally to form a single common channel; type III: urethral duplication, which occurs as part of partial or complete caudal duplication [2].

Despite various theories having been proposed regarding its embryological development, there is no single mechanism that can fully explain all subtypes of urethral duplication [6]. Diagnosis of urethral duplication is initially suggested by genital examination and subsequently confirmed through imaging techniques such as voiding cystourethrography (VCUG) or retrograde urethrography (RUG) [2,7]. The treatment approach is planned based on the type of duplication and the patient’s symptoms.

In this report, we present a case of urethral duplication detected perioperatively in a one-year-old boy and discuss the management of this anomaly.

## Case presentation

A one-year-old male presented to us in May 2025 with a request for subcoronal hypospadias repair. Physical examination and routine tests were unremarkable, and a tubularized incised plate urethroplasty (TIPU) was planned. At the start of surgery, a 5 Fr feeding catheter could not be advanced through the meatus, nor could a guidewire. Diagnostic cystourethroscopy was performed, revealing a blind-ending urethra at the penile level.

**Figure 1:**
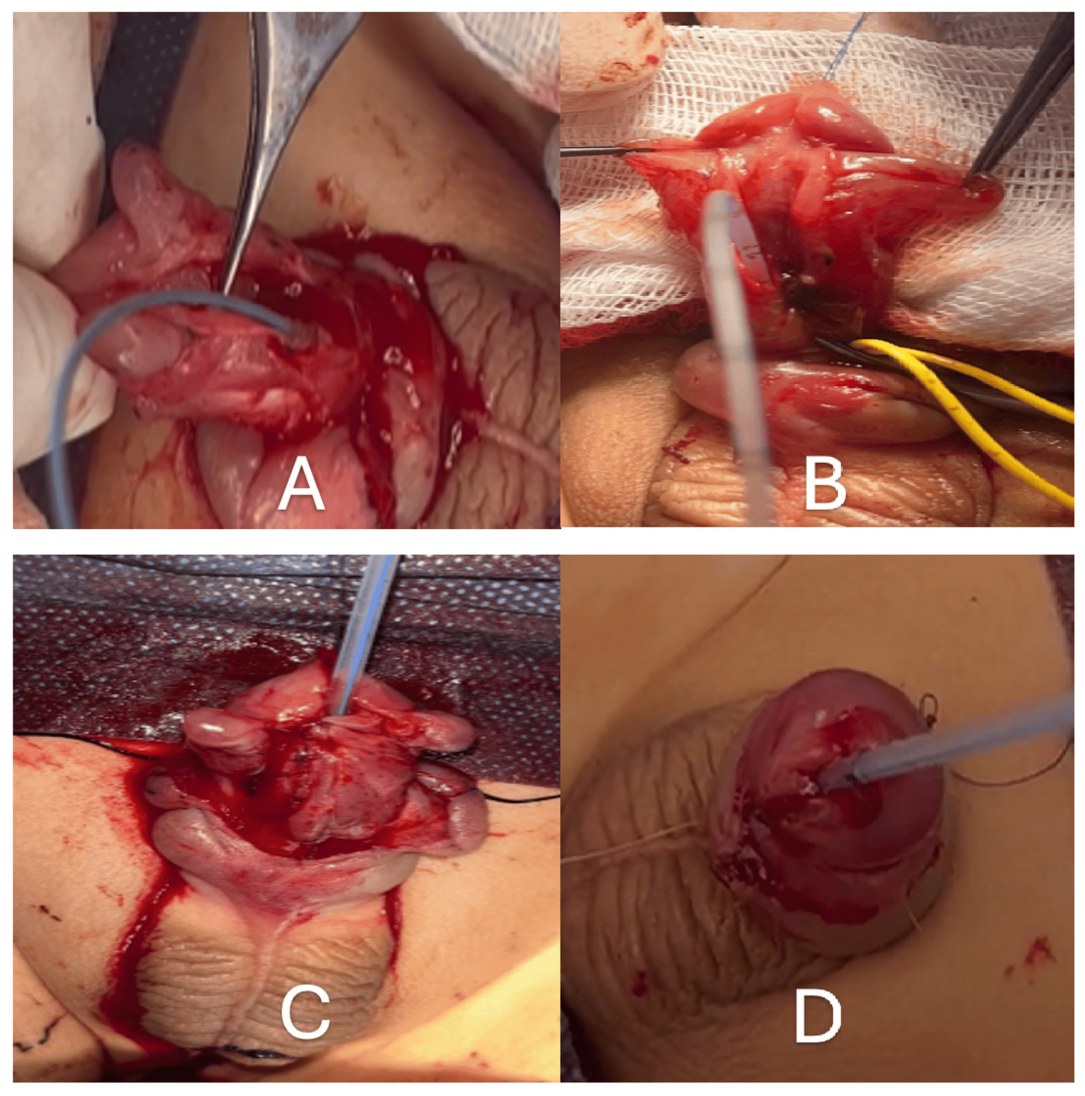
Surgical steps. (A) Guidewire placement through the accessory urethra. (B) Insertion of a feeding catheter into the real urethra. (C) Ventral view following augmentation urethroplasty using a preputial flap. (D) Postoperative appearance after skin closure. The surgical procedure was carried out in a stepwise manner. Initially, a guidewire was carefully advanced through the accessory urethra (A). This was followed by the insertion of a feeding catheter into the native urethra (B). Augmentation urethroplasty was then performed using a preputial flap, with the ventral aspect visualized intraoperatively (C). The procedure was completed with skin closure, and the immediate postoperative appearance was recorded (D).

**Figure 2:**
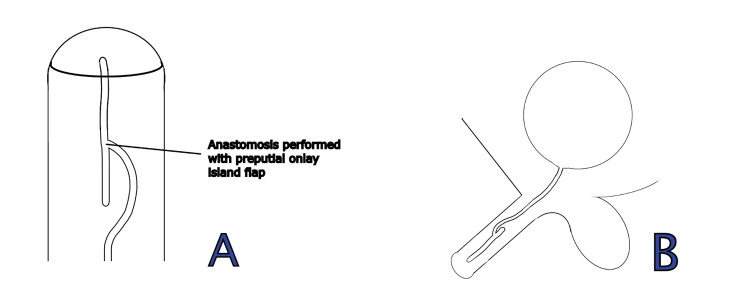
Schematic representation of the variant duplicated urethra anatomy in (A) axial and (B) sagittal planes. The schematic illustrates the anatomical configuration of the variant duplicated urethra in two planes. Panel A demonstrates the axial view, while panel B depicts the sagittal view, highlighting the spatial relationship between the native and accessory urethral channels.

Exploration with a ventral longitudinal incision exposed an accessory urethral channel slightly right of the midline. Urine flow from this channel was confirmed using the Crede maneuver, and successful catheterization was achieved, as shown in Figure 1. The anatomical variation is schematized in Figure 2. Cystourethroscopy via the accessory channel showed a normal prostatic urethra and bladder; no prostatic utricle was observed. It was determined that this channel served as the functional urethra.

An augmentation urethroplasty was performed using a preputial flap to connect the real and blind-ending urethras, followed by hypospadias repair. Ventral curvature (~20°) was corrected using a modified Nesbit technique. Anastomosis was done over a 6 Fr Foley catheter with 6-0 Vicryl sutures, followed by glanuloplasty and skin closure. The procedure was completed without complications.

**Figure 3:**
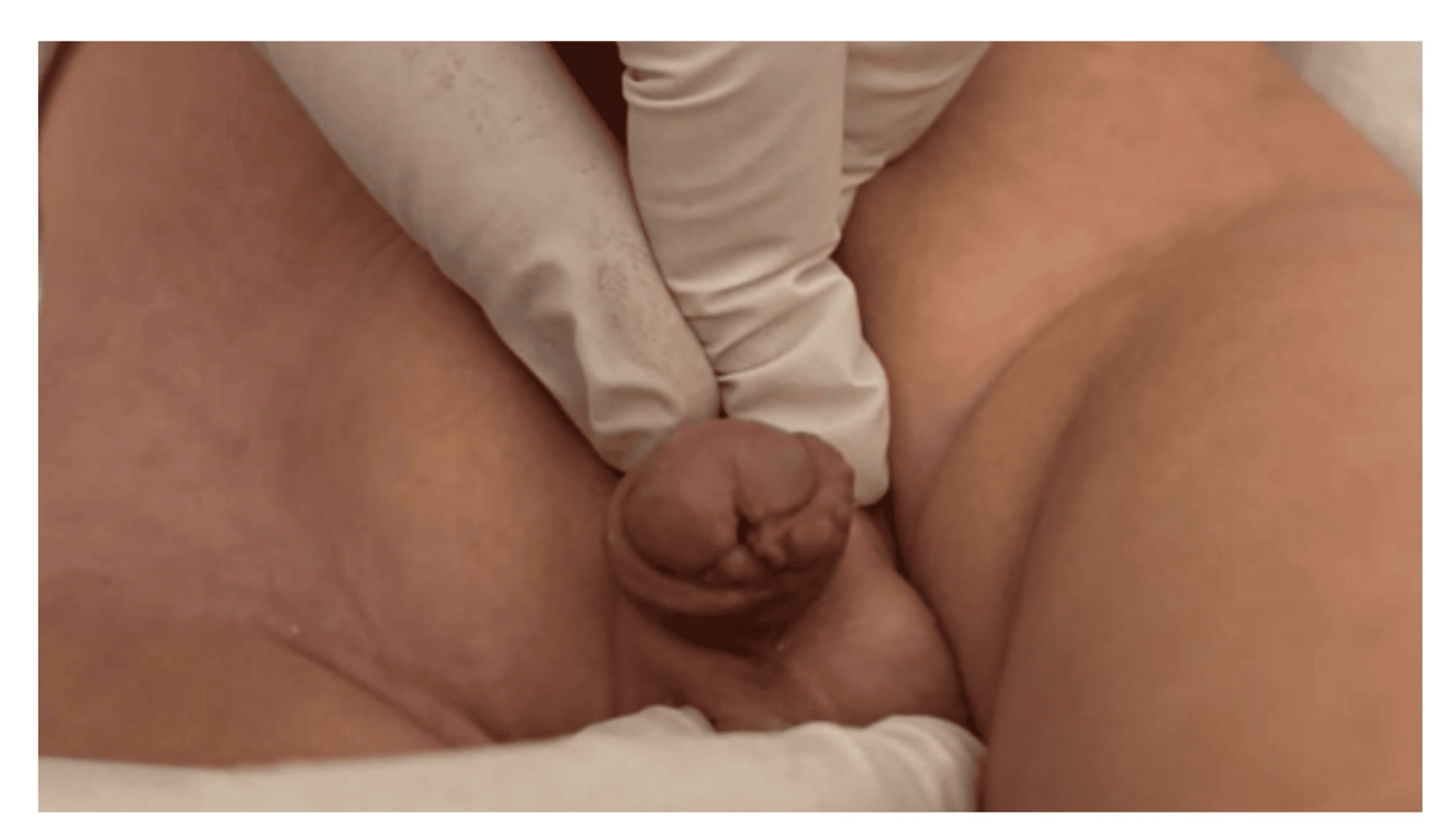
Postoperative image. At the second postoperative week, the catheter had been removed, and the surgical site appeared normal.

At the second postoperative week, the catheter had been removed, and the surgical site appeared normal, as demonstrated in Figure 3.

## Discussion

A review of the literature reveals that urethral duplications can be classified anatomically, such as sagittal and lateral duplications, or dorsal and ventral duplications, or based on their communication with each other and the bladder, as complete, incomplete, or blind-ending sinus duplications [2]. Most urethral duplications are dorsally located in the sagittal plane. These urethras are usually hypoplastic and have limited function, whereas the ventral urethra typically functions normally. From an anatomical perspective, sagittal duplications are typically categorized into three subtypes: epispadiac (dorsally located), hypospadiac (ventrally located), and Y-type duplications [8].

In our case, the ventral urethra ended blindly, and a duplicated urethra located to the right and dorsally to the ventral urethra was found to be connected to the bladder. It was also observed that the ventral urethra had a communication with the duplicated urethra proximally at the site of the blind-ending.

The most common complaint in individuals with urethral duplication is a double urinary stream. These patients may also present with dysuria, incontinence, or urinary tract infections. In our case, a one-year-old boy was brought to our clinic by his family due to distal hypospadias. Similar to our case, this condition may go unnoticed in children who are not yet toilet-trained and have only one visible urethral meatus.

Considering Effmann's classification, it can be said that our case represents a variant of type 1B duplicated urethra, according to the Effmann classification. In type 1B, there is a single external meatus, and the accessory urethra is connected to the ventral urethra. However, in this type, it is the ventral urethra that maintains continuity with the bladder. In contrast, in our case, the ventral urethra connects to the accessory urethra and then ends blindly, while the accessory urethra continues to the bladder [2].

Although rare, a duplicated urethra should be considered in children with hypospadias. A careful physical examination should assess the meatus location, presence of an accessory opening or fistula, and urine flow direction. In suspicious cases, catheterization during the exam may aid diagnosis. At the start of surgery, we routinely assess the urethra using a feeding catheter; if suspicion persists, VCUG or RUG can be performed, though urethroscopy remains the gold standard. Urethroscopy also helps identify additional anomalies such as the prostatic utricle or posterior urethral valve. Bladder and kidney ultrasonography is recommended to detect associated anomalies.

The treatment of urethral duplication should be tailored to the individual patient and the type of duplication. The most important factor in determining treatment is the presence of symptoms. If the patient is asymptomatic, no treatment may be necessary. This conservative approach is based on the principle of avoiding potential damage to neurovascular structures or the continence mechanism. Resection of the accessory urethra is recommended in patients who present with cosmetic complaints, discharge, urinary tract infections, or double urination [9]. In our case, the child was actually asymptomatic and was referred to us for subcoronal hypospadias. His blood and urine parameters, as well as his ultrasonography, were normal. During the procedure, cystoscopy revealed that the ventral urethra ended blindly, while the accessory urethra was in communication with the bladder. On cystourethroscopy via the accessory urethra, the prostatic urethra, bladder neck, and bladder were found to be normal. No prostatic utricle was observed.

The most commonly performed procedure in urethral duplication cases is surgical excision of the dorsal accessory urethra. However, there is no consensus regarding the most appropriate surgical approach. The choice of surgical technique is determined by the type of urethral duplication. In cases where the duplicated urethras terminate side by side at the glans, the septum separating the two meatuses can be excised to create a single meatus. More complex fistulas may require advanced urethroplasty techniques involving grafts or flaps [1]. In our case, we performed an anastomosis using a preputial flap between the ventral urethra, which opened to the external meatus, and the accessory urethra, which was connected to the bladder.

Savanelli et al. also used a prepuce onlay flap similar to our technique in a similar case in their published case report [10]. In such surgeries, the confluence of both urethras should be clearly identified, and if necessary, as we also practiced, augmentation should be performed to achieve an adequate caliber while preserving voiding function. Due to the risk of fistula formation, tissue interposition should be applied. Attention must be paid to the aesthetic and functional aspects of the meatus and glans.

Etensel et al. reported a 10-year-old boy with distal hypospadias in whom an abortive hypospadiatic duplicate urethra was identified and managed with TIPU. In their case, the ventral urethra maintained continuity with the bladder, while the accessory urethra ended blindly. In contrast, our case represents a rare variant, as the ventral urethra terminated blindly and the accessory urethra was the one communicating with the bladder. This difference highlights the wide anatomical variability of urethral duplications and underscores the importance of meticulous intraoperative evaluation for appropriate surgical planning [11].

Huang et al. described congenital prepubic sinus as a form of abortive hypospadiatic urethral duplication based on immunohistochemical analysis, supporting its classification as a dorsal urethral duplication variant. In their cases, the anomalous tract was represented by the accessory (dorsal) sinus, whereas in our case, the ventral urethra terminated blindly, and the accessory urethra maintained continuity with the bladder. This distinct anatomical presentation highlights the broad spectrum of urethral duplications and emphasizes the importance of detailed anatomical evaluation and individualized surgical planning for each patient [12].

A literature review shows that regarding the management of duplicated urethra, Arkan et al. reported a rare case of distal urethral duplication with a diverticular component in a girl aged five years and nine months. In their case, the urethral duplication was identified as a fistulous tract extending between the distal urethra and the clitoris and was surgically excised. In contrast, in our case, the ventral urethra terminated blindly, and the structure maintaining continuity with the bladder was the accessory urethra. This distinct presentation highlights that urethral duplications can occur in both sexes and underscores the need for careful consideration of such variations [13].

## Conclusions

Asymptomatic duplicated urethra cases were very rare but may be identified intraoperatively. In our case, during distal hypospadias surgery, a duplicated urethra was defined as a variant of type 1B. In addition to the hypospadias repair, augmentation urethroplasty was performed surgically. When performing distal hypospadias surgery, surgeons should not consider it solely as a distal procedure. These surgeries should be carried out in centers that are also capable of managing proximal and mid-penile hypospadias repairs.
